# LPS Stimulation Induces Small Heterodimer Partner Expression Through the AMPK-NRF2 Pathway in Large Yellow Croaker (*Larimichthys crocea*)

**DOI:** 10.3389/fimmu.2021.753681

**Published:** 2021-11-08

**Authors:** Jianlong Du, Xiaojun Xiang, Dan Xu, Kun Cui, Yuning Pang, Wei Xu, Kangsen Mai, Qinghui Ai

**Affiliations:** ^1^ Key Laboratory of Aquaculture Nutrition and Feed (Ministry of Agriculture and Rural Affairs) & Key Laboratory of Mariculture (Ministry of Education), Ocean University of China, Qingdao, China; ^2^ Laboratory for Marine Fisheries Science and Food Production Processes, Qingdao National Laboratory for Marine Science and Technology, Qingdao, China

**Keywords:** SHP, AMPK, NRF2, large yellow croaker, LPS

## Abstract

The mall heterodimer partner (SHP) plays an important regulatory role in mammal inflammation. The main objective of this study was to investigate the response of SHP to inflammatory stimulation and its underlying mechanism. The *shp* gene from large yellow croakers, was cloned, and this gene is mainly expressed in the liver and intestine. Lipopolysaccharide (LPS) stimulation induced the mRNA expression and protein level of SHP in macrophages of large yellow croakers. Overexpression of SHP significantly decreased mRNA expression of *tnfα*, *il-1β*, *il-6* and *cox2* induced by LPS treatment in macrophages. LPS stimulation increased the phosphorylation level of Adenosine 5’-monophosphate (AMP)-activated protein kinase (AMPK) in macrophages. AMPK inhibitor treatment significantly decreased the expression of SHP induced by LPS while AMPK activator significantly increased the expression of SHP. The nuclear factor-erythroid 2-related factor 2 (NRF2) increased the promoter activity of SHP in large yellow croakers and the level of nuclear NRF2 was increased by LPS stimulation and AMPK activation. NRF2 inhibitor treatment significantly decreased mRNA expression of *shp* induced by LPS and AMPK activator. In conclusion, LPS can induce SHP expression by activating the AMPK-NRF2 pathway while SHP could negatively regulate LPS-induced inflammation in large yellow croakers. This study may be benefit to the development of immunology of marine fish and provide new ideas for inflammation-related diseases.

## Introduction

Inflammation is a physiological response of the body to harmful stimuli and an innate immunity mechanism that maintains cellular homeostasis (1). The small heterodimer partner (SHP) is a crucial regulator in the inflammation system (2–4). Loss of SHP function enhances lipopolysaccharide (LPS)-induced expression of pro-inflammatory cytokines and increases liver injury in mice (4, 5). However, studies in other mammals showed different changes of SHP in response to inflammatory stimulation. Thus, understanding the response of SHP to inflammatory stimulation and the underlying mechanism needs additional study.

SHP was originally cloned in humans and mice in 1996 (6). Subsequent studies showed that SHP is expressed in various tissues and is predominantly expressed in the gallbladder and liver (7, 8). Mammal studies demonstrated that SHP is an orphan member of the nuclear hormone receptor superfamily which lacks the conserved DNA-binding domain (DBD) (6). As a transcriptional corepressor, SHP participates in regulating expression of genes involved in immune response, glucose, lipid and bile acid metabolism through direct binding to several nuclear receptors (9, 10). The teleosts are the largest and oldest vertebrate group on the earth. Determination of the physiological function of SHP in teleosts will help reveal its evolution in vertebrates. Little is known about SHP in fish, but studies on *Oreochromis niloticus* and *Oncorhynchus mykiss* show that SHP is highly expressed in the liver and intestine (11, 12). However, the function and regulation of SHP in fish remains unknown.

The large yellow croaker (*Larimichthys crocea*) is one of the most economically important marine fish and is widely cultured in China. Based on the established method of cell culture and a large number of immunity studies (13–15), the large yellow croaker can be used as a model for studying the molecular mechanisms of inflammation in teleosts. This study explored the role of SHP in response to inflammation in the large yellow croaker.

## Methods

### Cloning and Sequence Analysis

The cDNA of large yellow croaker *shp* was cloned according to a method described previously (16). SMARTer™ RACE cDNA Amplification Kit (Clontech, USA) was used to clone the 3′- and 5′- end sequence. Then amino acid sequences in SHP were deduced from the full-length cDNA sequence. The molecular weight and isoelectric point were calculated by a Compute pI/Mw tool (http://web.expasy.org/compute_pi/). The multiple sequence alignment was performed using DNAman (Lynnon BioSoft, Canada). The phylogenetic analyses were obtained by the neighbor-joining method and the tree was constructed using the MEGA7 program (https://www.megasoftware.net/).

### Tissue Distribution and Subcellular Localization

The mRNA expression of *shp* was determined in the heart, liver, kidney, brain, adipose tissue, intestine, eye, gill, muscle and spleen of large yellow croakers. The fish with weight of 300-400g were obtained from Xiangshan Harbor Nusery Co., Ltd. (Ningbo, China). Tissues were collected from 6 individuals and distributed into three samples. Samples were rapidly frozen in liquid nitrogen and stored at −80°C.

Large yellow croaker SHP-GFP and lamin B-RFP fusion protein were constructed to determine the subcellular localization. The open reading frame of croaker SHP was introduced into vector pcDNA 3.1-EGFP (17). HEK 293T cells (5 × 10^5^ cells/mL) were transfected with GFP, SHP-GFP and lamin B-RFP expression plasmid. After the cells were cultured for 36 h, they were fixed in 4% paraformaldehyde before observing the localization through a laser confocal microscopy (Leica, Germany).

### Cell Culture and Treatment

Macrophage cell lines of the large yellow croaker (LCM) cells (13) were cultured in DMEM/F12 supplemented with 15% fetal bovine serum (FBS) (Biological Industries, Israel), 100 U/mL penicillin and 100 μg/mL streptomycin at 27°C and 5% CO_2_. HEK 293T cells were cultured in high-glucose DMEM supplemented with 10% FBS, 100 U/mL penicillin and 100 μg/mL streptomycin at 37°C and 5% CO_2_.

To investigate the response of SHP to LPS stimulation, LCM cells were seeded into 6-well plate (1 × 10^6^ cells) treated with 0, 25, 50 and 100 μg/mL LPS for 6 h. To explore the role of SHP in inflammatory regulation, LCM cells were injected with recombinant adenovirus encoding croakers SHP (advSHP) for 36 h and then stimulated with LPS (50 μg/mL, Sigma, USA) for 0, 0.25, 0.5, 1, 2 and 4 h. In addition, to study the effect of adenosine monophosphate (AMP)-activated protein kinase (AMPK) on the expression of SHP, AICAR (500 µM, AMPK agonist, MCE, USA) was used to treat LCM cells for 3 h. Cells were pretreated with CC (5 µM, Compound C, AMPK inhibitor, MCE) for 1 h and then stimulated with LPS for 3 h. To investigate the effect of nuclear factor-erythroid 2-related factor 2 (NRF2) on the expression of SHP, cells were pretreated with ML385 (5 µM, NRF2 inhibitor, MCE) for 1 h and then stimulated with LPS (50 μg/mL) or AICAR (500 µM) for 3 h.

### Quantitative Real-Time PCR

The mRNA expression of genes was analyzed by quantitative real-time PCR as described by Du et al. (18). Specific primers were designed to detect the expression of cyclooxygenase 2 (*cox2*), interleukin 1 beta (*il-1β*), interleukin 6 *(il6*) and small heterodimer partner (*shp*). β-actin and glyceraldehyde-3-phosphate dehydrogenase (*gapdh*) were used as housekeeping genes. All primer sequences are listed in **Table 1**. The levels of gene expression were calculated and normalized *via* the 2^−ΔΔCT^ method (19).

**Table 1:** Primers used for qPCR analysis.

Genes	Forward Sequences (5′–3′)	Reverse Sequences (5′–3′)
*cox2*	CTGGAAAGGCAACACAAGC	CGGTGAGAGTCAGGGACAT
*il-1β*	AGCCAATCTGGCAAGGATCA	GCTGATGAACCAGTTGTTGT
*il6*	CGACACACCCACTATTTACAAC	TCCCATTTTCTGAACTGCCTCT
*tnfa*	CGTCCTGGTGTTTGCTTGGT	TGTTTTCTCGGCAGTCGTCTT
*shp*	GCGACGGACAGTGTGCTTGAA	ACTGGTCGTTTGGTGGCATCTG
*gapdh*	GACAACGAGTTCGGATACAGC	CAGTTGATTGGCTTGTTTGG
*β-actin*	CTACGAGGGTTATGCCCTGCC	TGAAGGAGTAACCGCGCTCTG

cox2, cyclooxygenase 2; il-1β, interleukin 1 beta; il6, interleukin 6; shp, small heterodimer partner; GAPDH, glyceraldehyde-3-phosphate dehydrogenase; tnfa, tumor necrosis factor alpha.

### Western Blotting

Western blot experiments were performed as described by Tan et al. (20). Primary antibodies used in this study were against SHP (OM107591, Omnimabs, USA), FLAG (14793, CST, USA), phosphor-AMPKα (Thr172) (2531, CST, USA), phosphor-ACC (Ser79) (3661, CST, USA), NRF2 (ab62352, Abcam, USA), H3 (4499, CST, USA) and GAPDH (309154, ZSGB-Bio, China). The immunoreactive protein was visualized using an electrochemiluminescence kit (Beyotime, China) and scanned by an Epson Perfection V33 scanner.

### Luciferase Reporter Assay

The promoter of large yellow croaker SHP was cloned into the reporter plasmid (pGL3-basic vector, Promega, USA) by a ClonExpress II One Step Cloning Kit (Vazyme, China) (17). The expression plasmid of large yellow croaker NRF2 was stored in our laboratory. HEK 293T cells were seeded into 24-well plate (5 × 10^5^ cells) and co-transfected with reporter plasmids (200 ng/well), phRL-CMV plasmid (20ng/well, Promega, USA), and expression plasmids (200-600 ng/well). After 24 h transfection, cells were collected and the luciferase activity was measured using a TransDetect double-luciferase reporter assay kit (TransGen Biotech, China).

### Statistical Analysis

Data are presented as means ± SEMs and were analyzed using one-way ANOVA, followed by Tukey’s Test. The software used was SPSS 20.0 (SPSS, USA). The significance of the difference between two groups was determined by Student’s t-tests. A *P* < 0.05 was considered to be statistically significant.

## Results

### Molecular Cloning and Phylogenetic Analysis

**Figure 1:**
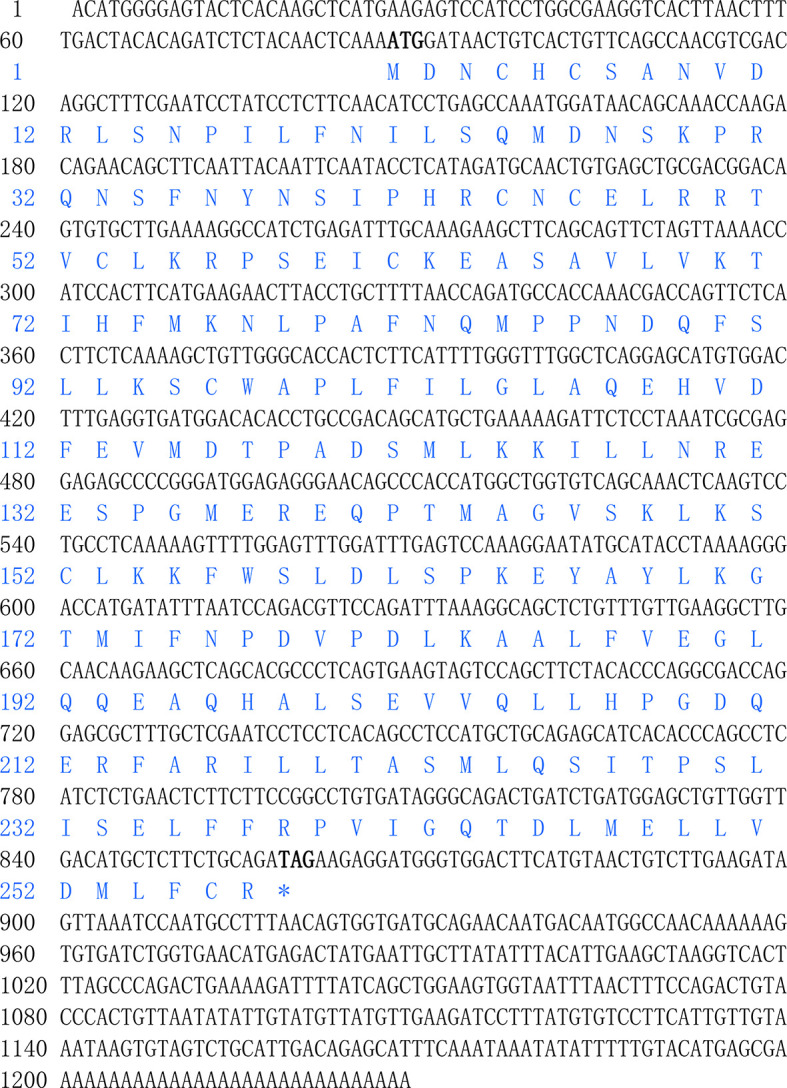
Nucleotide and deduced amino acid sequences of SHP of large yellow croaker.

Full-length cDNA of SHP (GenBank Acc. No. KY745777) comprised 1228 bp with the following features: 86 bp 5′-untranslated region (UTR), 774 bp open reading frame (ORF) and 368 bp 3′-UTR. The ORF encodes a polypeptide of 258 amino acids with a predicted molecular weight of 29.25 KDa and theoretical isoelectric point of 6.31 **(** **Figure 1** **)**.

**Figure 2:**
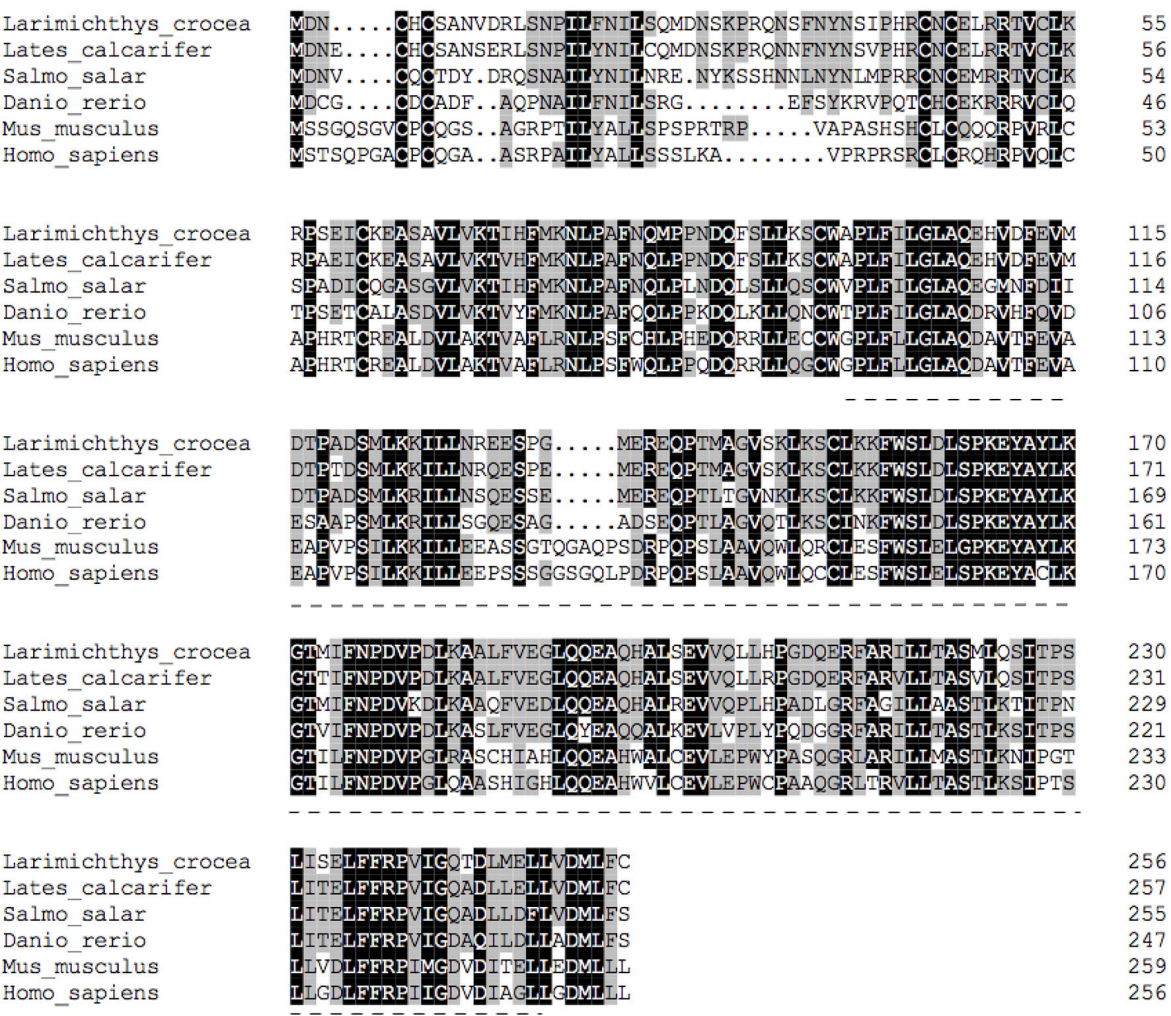
Multiple sequence alignment of SHP with deduced amino acid sequences of other fish or mammal animals. Accession numbers used are: *Lates calcarifer* (XP_018541889.1), *Salmo salar* (NP_001134122.1), *Danio rerio* (NP_001243120), *Mus musculus* (NP_035980.1) and *Homo sapiens* (NP_068804.1).

**Figure 3:**
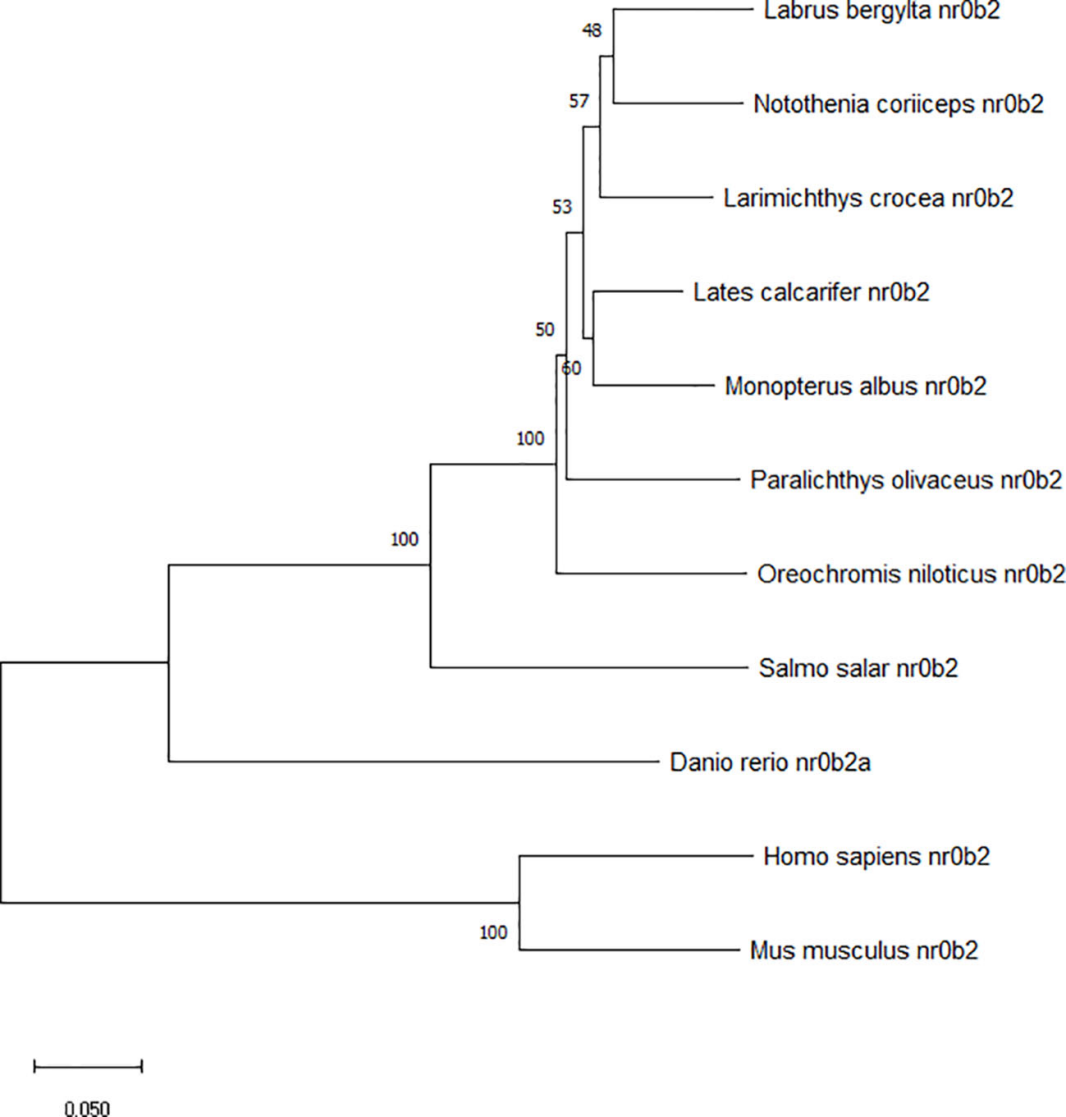
Phylogenetic tree of large yellow croaker SHP with other vertebrate counterparts. The horizontal branch length is proportional amino acid substitution rate per site. The numbers represent the frequencies with which the tree topology presented here was replicated after 1000 bootstrap iterations.

The deduced amino acid sequence of the SHP polypeptide in the large yellow croaker, exhibited the highest (89.9%) identity with Barramundi (*Lates calcarifer*), followed by Atlantic salmon (*Salmo salar*) (78.3%) and Zebrafish (*Danio rerio*) (75.91%) **(** **Figure 2** **)**. Phylogenetic analysis clustered large yellow croaker SHP with other teleosts and mammals **(** **Figure 3** **)**.

### Tissue Distribution and Localization of SHP

**Figure 4:**
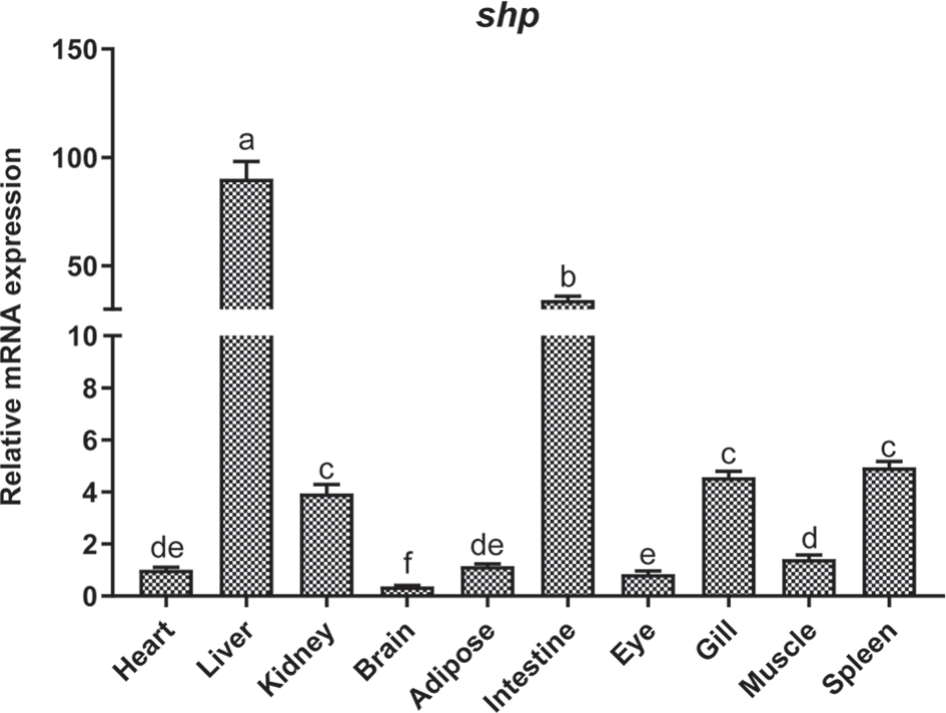
Tissue distribution of SHP in large yellow croakers. Data are presented as means ± SEMs, n = 3 replicate experiments. Significance was evaluated by one-factor ANOVA followed by Duncan’s multiple range test. Labeled means without a common letter differ, *P* < 0.05.

The mRNA expression of *shp* was determined in different tissues of the large yellow croaker such as heart, liver, head kidney, brain, adipose tissue, intestine, eye, gill, muscle and spleen. The highest expression of *shp* was observed in liver and intestine. The kidney, Gill and spleen had moderate levels while the expression in the brain was lowest (*P* < 0.05) **(** **Figure 4** **)**.

**Figure 5:**
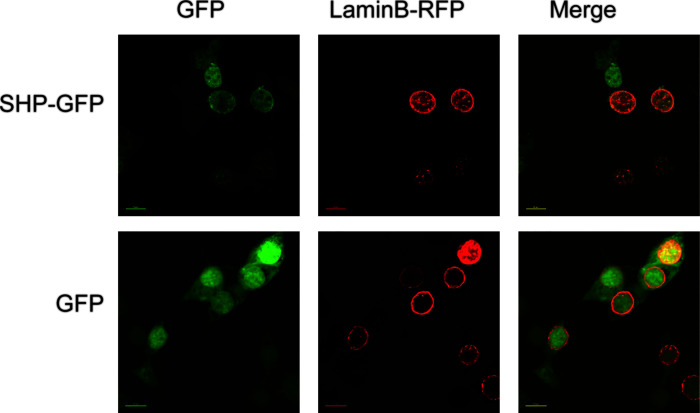
Localization of large yellow croaker SHP in HEK 293T cells. GFP, Green fluorescent protein; SHP-GFP, small heterodimer partner and GFP fusion proteins; Lamin B-RFP, Lamin B and red fluorescent protein fusion proteins.

The subcellular localization analysis indicated that SHP-GFP fusion protein in HEK 293T cells could target the nucleus. In contrast, GFP alone was present in the whole cell **(** **Figure 5** **)**.

### LPS Stimulation Induces Expression of SHP

**Figure 6:**
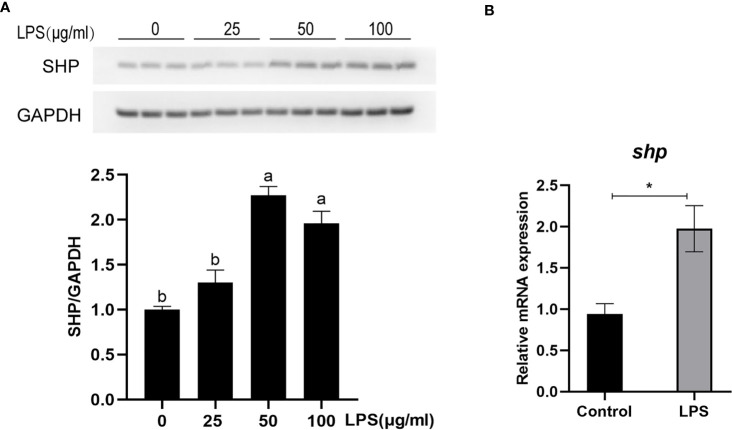
LPS induces the expression of SHP in large yellow croakers. **(A)** Immunoblots for SHP in macrophages after LPS stimulation. **(B)** Gene expression of *shp* in macrophages after LPS stimulation. Data are presented as means ± SEMs, n = 3 replicate experiments. Significance was evaluated by one-factor ANOVA followed by Duncan’s multiple range test. Labeled means without a common letter differ, *P* < 0.05. ^∗^Different from control, *P* < 0.05 (Student’s *t*-test). LPS, lipopolysaccharide; SHP, small heterodimer partner; GAPDH, glyceraldehyde-3-phosphate dehydrogenase.

The protein level and mRNA expression of SHP was detected in macrophages after LPS stimulation. A 50 and 100 μg/ml LPS stimulation significantly induced the protein level of SHP (*P* < 0.05) **(** **Figure 6A** **)**. LPS treatment significantly increased the mRNA expression of *shp* in macrophages (*P* < 0.05) **(** **Figure 6B** **)**.

### Overexpression of SHP Inhibits LPS-Induced Expression of Pro-Inflammatory Genes

**Figure 7:**
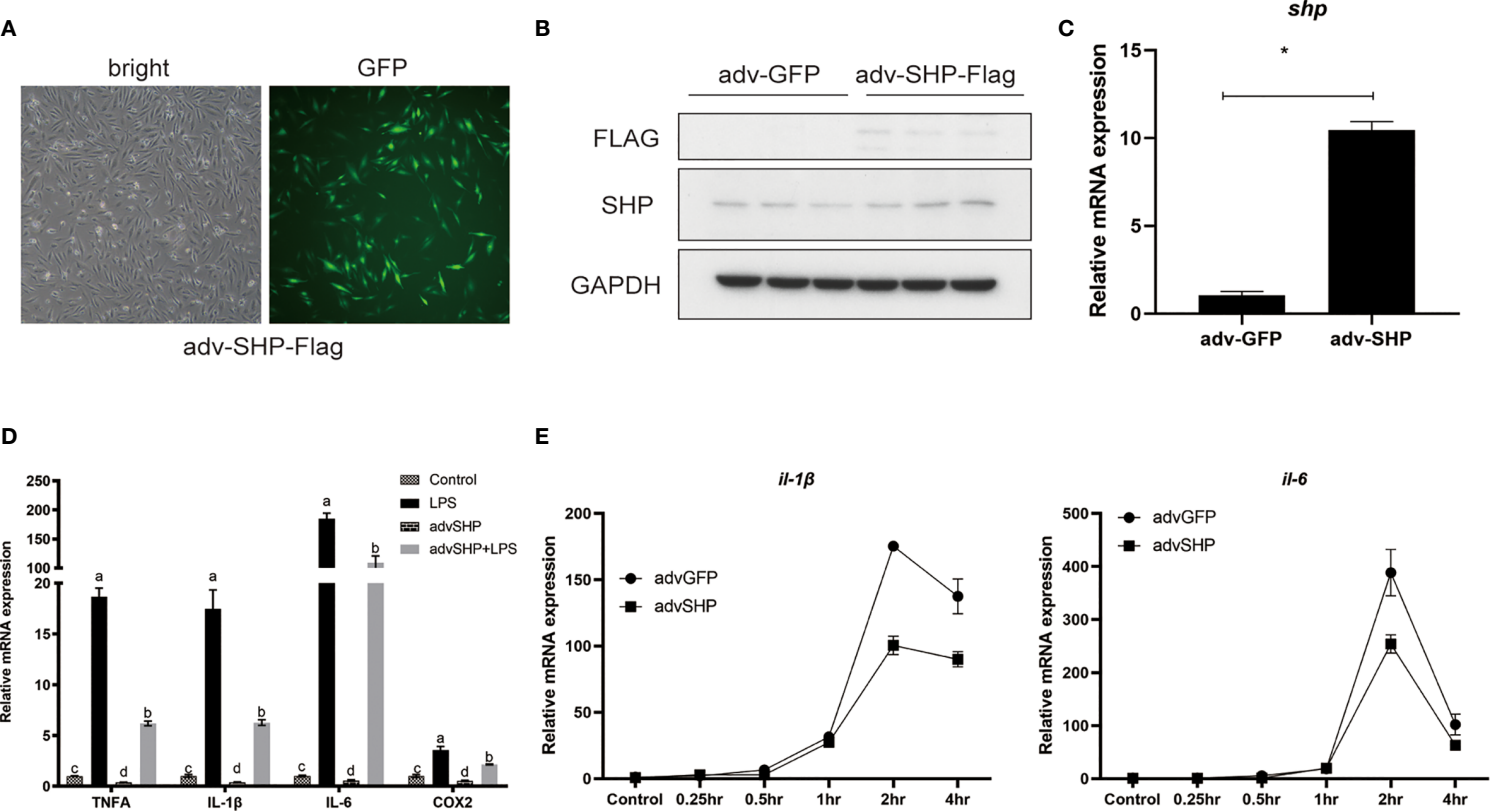
Effects of SHP on LPS-induced inflammation of large yellow croakers. **(A-C)** Fluorescence analysis, immunoblots and qRT-PCR assays for SHP in macrophages infected by croaker SHP adenovirus. **(D)** Expression of *tnfα, il-1β, il-6* and *cox2* in macrophages after LPS stimulation alone or with SHP overexpression. **(E)** Expression of *il-1β* and *il-6* in macrophages after LPS stimulation at different time points. Data are presented as means ± SEMs, n = 3 replicate experiments. Significance was evaluated by one-factor ANOVA followed by Duncan’s multiple range test. Labeled means without a common letter differ, *P* < 0.05. ^∗^Different from control (adv-GFP), *P* < 0.05 (Student’s *t*-test). *cox2*, cyclooxygenase 2; LPS, lipopolysaccharide; *il-1β*, interleukin 1 beta; *il6*, interleukin 6; SHP, small heterodimer partner; GAPDH, glyceraldehyde-3-phosphate dehydrogenase; *tnfa*, tumor necrosis factor alpha.

To study the role of SHP in response to LPS stimulation, macrophages were infected with recombinant adenovirus encoding croakers SHP **(** **Figure 7A** **)**. SHP recombinant adenovirus significantly increased the protein level and mRNA expression of SHP in macrophages (*P* < 0.05) **(** **Figures 7B, C** **)**. LPS treatment also significantly increased the mRNA expression of pro-inflammatory genes, such as *tnfα*, *il-1β*, *il-6* and *cox2* (*P* < 0.05) **(** **Figure 7D** **)**. Overexpression of SHP significantly decreased mRNA expression of *tnfα*, *il-1β*, *il-6* and *cox2* induced by LPS treatment in macrophages (*P* < 0.05) (**Figure 7D**). To confirm this effect, we detected the expression of *il-1β* and *il-6* in macrophages after LPS treatment at different time points. Increases in the expression of *il-1β* and *il-6* were sustained up to 2 h, followed by a drop at 4 h **(** **Figure 7E** **)**. SHP overexpression decreased mRNA expression of *il-1β* and *il-6* at 2 h and 4 h (**Figure 7E**).

### LPS Induces SHP Expression Through the AMPK Pathway

**Figure 8:**
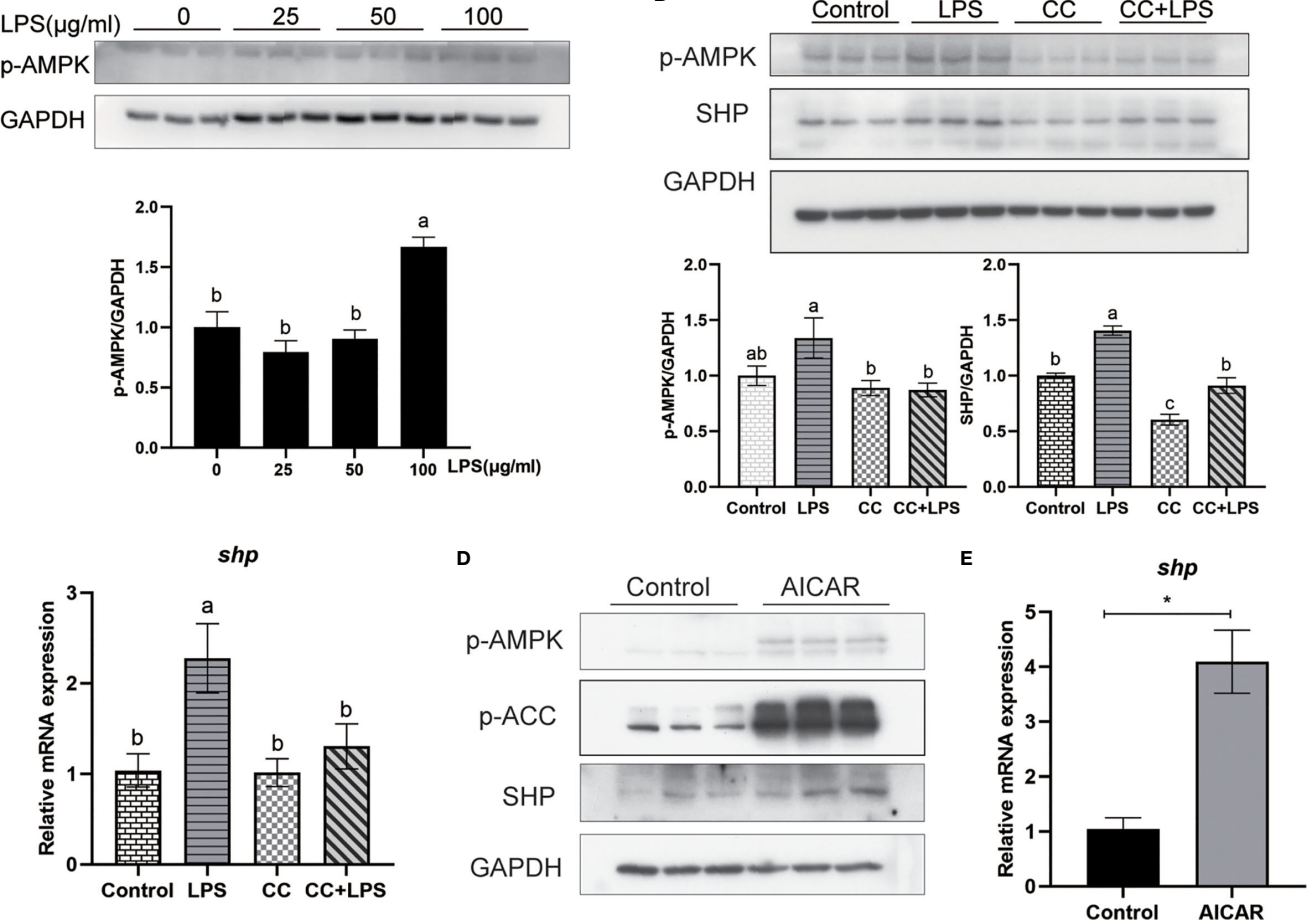
LPS induces the expression of SHP through the AMPK signaling pathway. **(A)** Immunoblots for phosphorylated AMPK in macrophages after LPS stimulation. **(B)** Immunoblots for phosphorylated AMPK and SHP in macrophages after LPS stimulation alone or with AMPK inhibitor (CC). **(C)** Expression of *shp* in macrophages after LPS stimulation or AMPK inhibitor treatment. **(D)** Effects of AMPK activator (AICAR) on protein levels of phosphorylated AMPK, phosphorylated ACC and SHP in macrophages. **(E)** Gene expression of *shp* in macrophages after treatment with AICAR. Data are presented as means ± SEMs, n = 3 replicate experiments. Significance was evaluated by one-factor ANOVA followed by Duncan’s multiple range test. Labeled means without a common letter differ, *P* < 0.05. ^∗^Different from control, *P* < 0.05 (Student’s *t*-test). ACC, Acetyl-CoA carboxylase; AMPK, Adenosine 5’-monophosphate (AMP)-activated protein kinase; AICAR, 5-aminoimidazole-4-carboxamide ribonucleotide; CC, Compound C; LPS, lipopolysaccharide; SHP, small heterodimer partner; GAPDH, glyceraldehyde-3-phosphate dehydrogenase.

We studied how LPS induced the expression of SHP. LPS induced the expression of SHP, and a similar trend was obtained in the phosphorylation level of AMPK. LPS treatment significantly increased the phosphorylation level of AMPK in macrophages (*P* < 0.05) **(** **Figure 8A** **)**. The AMPK inhibitor (CC) treatment significantly decreased the phosphorylation level of AMPK and protein level of SHP induced by LPS (*P* < 0.05) **(** **Figure 8B** **)**. Similarly, CC treatment decreased LPS-induced mRNA expression of *shp* (*P* < 0.05) **(** **Figure 8C** **)**. AICAR, the AMPK activator, significantly increased the phosphorylation of AMPK and ACC **(** **Figure 8D** **)**, and increased the protein level and mRNA expression of SHP (*P* < 0.05) **(** **Figures 8D, E** **)**.

### NRF2 Is Involved in the Effect of LPS and AMPK Pathway on SHP

**Figure 9:**
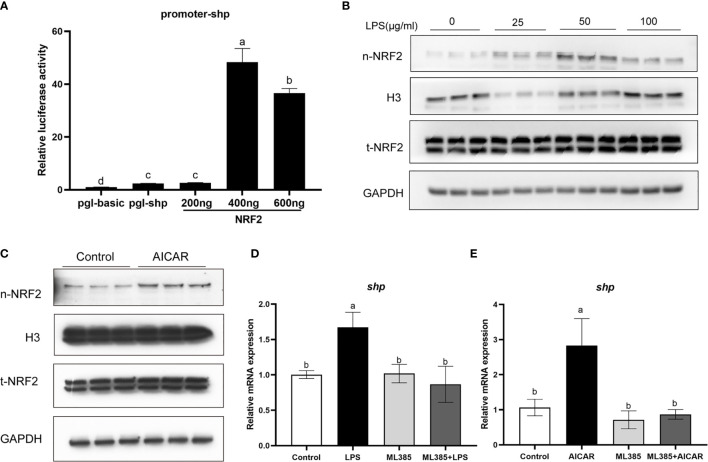
NRF2 is involved in the effect of LPS and AMPK pathway on SHP. **(A)** Relative luciferase activities of SHP promoter of large yellow croaker in HEK 293T cells after NRF2 overexpression. **(B)** Immunoblots for nuclear NRF2 in macrophages after LPS stimulation. **(C)** Immunoblots for nuclear NRF2 in macrophages after treatment with AMPK activator (AICAR). **(D)** Expression of *shp* in macrophages after treatment with LPS and NRF2 inhibitor (ML385). **(E)** Expression of *shp* in macrophages after treatment with AICAR and ML385. Data are presented as means ± SEMs, n = 3 replicate experiments. Significance was evaluated by one-factor ANOVA followed by Duncan’s multiple range test. Labeled means without a common letter differ, *P* < 0.05. AICAR, 5-aminoimidazole-4-carboxamide ribonucleotide; LPS, lipopolysaccharide; SHP, small heterodimer partner; GAPDH, glyceraldehyde-3-phosphate dehydrogenase. NRF2, nuclear factor-erythroid 2-related factor 2.

Overexpression of large yellow croaker NRF2 significantly increased the promoter activity of SHP in HEK293T cells (*P* < 0.05) **(** **Figure 9A** **)**. In macrophages, LPS stimulation increased the level of nuclear NRF2 **(** **Figure 9B** **)**. In addition, the level of nuclear NRF2 was increased in macrophages after treatment with AICAR **(** **Figure 9C** **)**. To confirm the role of NRF2 in the effect of LPS and AMPK on SHP, macrophages were treated with NRF2 inhibitor (ML385), AICAR or LPS. The ML385 treatment significantly decreased mRNA expression of *shp* induced by LPS and AICAR (*P* < 0.05) **(** **Figures 9D, E** **)**.

## Discussion

In the present study, we found that SHP is widely expressed in tissues of the large yellow croaker and highly expressed in the liver and intestine. These results are consistent with studies on *Oreochromis niloticus* and *Oncorhynchus mykiss* (11, 12). The extensive expression of *shp* in different tissues indicated that SHP may have a variety of physiological functions in teleost fish. The analysis of the deduced amino acid sequence of SHP showed that, as in mammals (6), the DNA-binding domain was also lacking in large yellow croakers. Subcellular localization analysis indicated that SHP of large yellow croaker could target the nucleus. In addition, we previously detected that SHP could bind to a transcription factor nuclear factor-kB p65 in large yellow croakers (17). These findings correspond with studies on mammals showing that SHP is a transcriptional repressor of gene expression by directly binding to a variety of nuclear receptors (21–24). These results suggest that the function of SHP in transcriptional regulation has been evolutionarily conserved in teleosts.

LPS stimulation induced the mRNA expression and protein level of SHP in macrophages of large yellow croakers, which indicates that SHP may be involved in the regulation of LPS-induced inflammation. Then, we studied the role of large yellow croaker SHP in the regulation of inflammation. Results showed that overexpression of SHP inhibited the inducing effect of LPS on mRNA expression of pro-inflammatory genes such as *tnfα*, *il-1β*, *il-6* and *cox-2*. Our previous study confirmed that large yellow croaker SHP could bind to the p65 protein, a key regulator of inflammation, and suppress its transcriptional activity (17). Consistent with previous studies in mammals (2, 4, 25), present results indicated that SHP can negatively regulate inflammatory responses in fish which suggested that the anti-inflammation exerted by SHP and the molecular mechanism may be relatively conservative.

Because SHP has a role in regulating inflammation, we studied how SHP expression is regulated after LPS stimulation. AMPK is known to be a negative mediator of inflammation (26). Mammal studies showed that treatment with AMPK activating drugs induced the expression of SHP (27, 28). We investigated whether LPS could affect expression of SHP by AMPK. Results showed LPS stimulation induced the phosphorylation level of AMPK in macrophages of large yellow croakers. AICAR mediated-activation of AMPK increased expression of SHP, and inhibiting AMPK by CC decreased LPS induced-expression of SHP. These results indicated that AMPK was involved in the increase of SHP induced by LPS. In mammals, NRF2 is a positive transcriptional regulator of SHP (29). The activation of NRF2 with phosphorylation by AMPK results in its nuclear accumulation (30). Consistent with mammal studies, we found that NRF2 was a regulator of SHP in large yellow croakers, while AMPK activation and LPS stimulation increased the level of nuclear NRF2. Inhibition of NRF2 suppressed the expression of SHP induced by AMPK activation and LPS stimulation. These results suggest that LPS may induce SHP expression by activating the AMPK-NRF2 pathway in large yellow croakers. In addition, a mouse study showed that the AMPK-USF1 pathway also contributes to LPS-induced expression of SHP (4). However, not all studies have reached this conclusion. Some research showed that LPS stimulation inhibited the phosphorylation level of AMPK and decreased the expression of SHP (31). This may be caused by the different changes of AMPK in response to immune stimulation (32–35), which needs further study.

In conclusion, we found that SHP plays an important role in the negative regulation of LPS-meditated inflammation in large yellow croakers. LPS can induce SHP expression by activating the AMPK-NRF2 pathway. Our results provide new ideas and enrich the basic research on immunology of marine fish, and SHP may be an effective target for regulating inflammation in large yellow croakers.

## Data Availability Statement

The datasets presented in this study can be found in online repositories. The names of the repository/repositories and accession number(s) can be found below: GenBankAcc.No. KY745777 https://www.ncbi.nlm.nih.gov/nuccore/KY745777.1/.

## Ethics Statement

The animal study was reviewed and approved by Institutional Animal Care and Use Committee of Ocean University of China.

## Author Contributions

JD, QA, WX, and KM designed the research. JD conducted the experiments and sample analyses with the help of XX, DX, KC, and YP. JD and QA analyzed the data. JD and QA wrote the paper. All authors read and approved the final manuscript.

## Funding

This study was supported by the National Science Fund for Distinguished Young Scholars of China (grant no. 31525024), the Key Program of National Natural Science Foundation of China (grant no. 31830103), the Scientific and Technological Innovation of Blue Granary (grant no. 2018YFD0900402), the Ten-thousand Talents Program (grant no. 2018-29) and the China Postdoctoral Science Foundation (grant no.2019M662449).

## Conflict of Interest

The authors declare that the research was conducted in the absence of any commercial or financial relationships that could be construed as a potential conflict of interest.

The reviewer JX declared a shared affiliation with all authors to the handling editor at the time of review.

## Publisher’s Note

All claims expressed in this article are solely those of the authors and do not necessarily represent those of their affiliated organizations, or those of the publisher, the editors and the reviewers. Any product that may be evaluated in this article, or claim that may be made by its manufacturer, is not guaranteed or endorsed by the publisher.
